# Supplementary material for: Macrophage inflammation resolution requires CPEB4-directed offsetting of mRNA degradation
Source: eLife. 2022 Apr 20;11:e75873. doi: 10.7554/eLife.75873 (PMC9094754; doi:10.7554/eLife.75873)

Black Box. Figure 2B. CPEB4.
Red Boxes. Figure 2 - figure supplement 1. Pannel A. CPEB4. Replicates 1 and 2

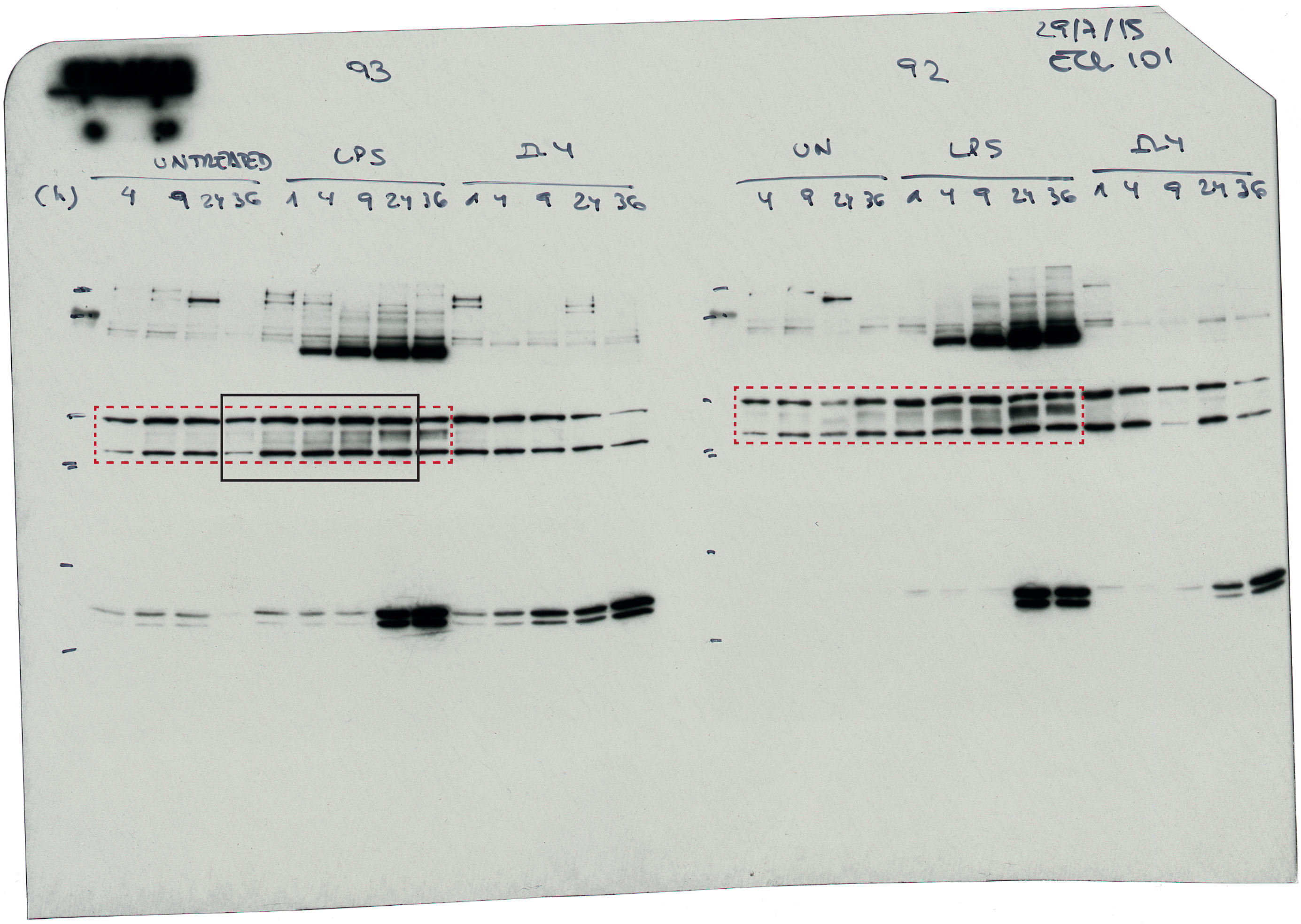

Black Box. Figure 2B. CPEB4.
Red Boxes. Figure 2 - figure supplement 1. Pannel A. Tubulin. Replicates 1 and 2

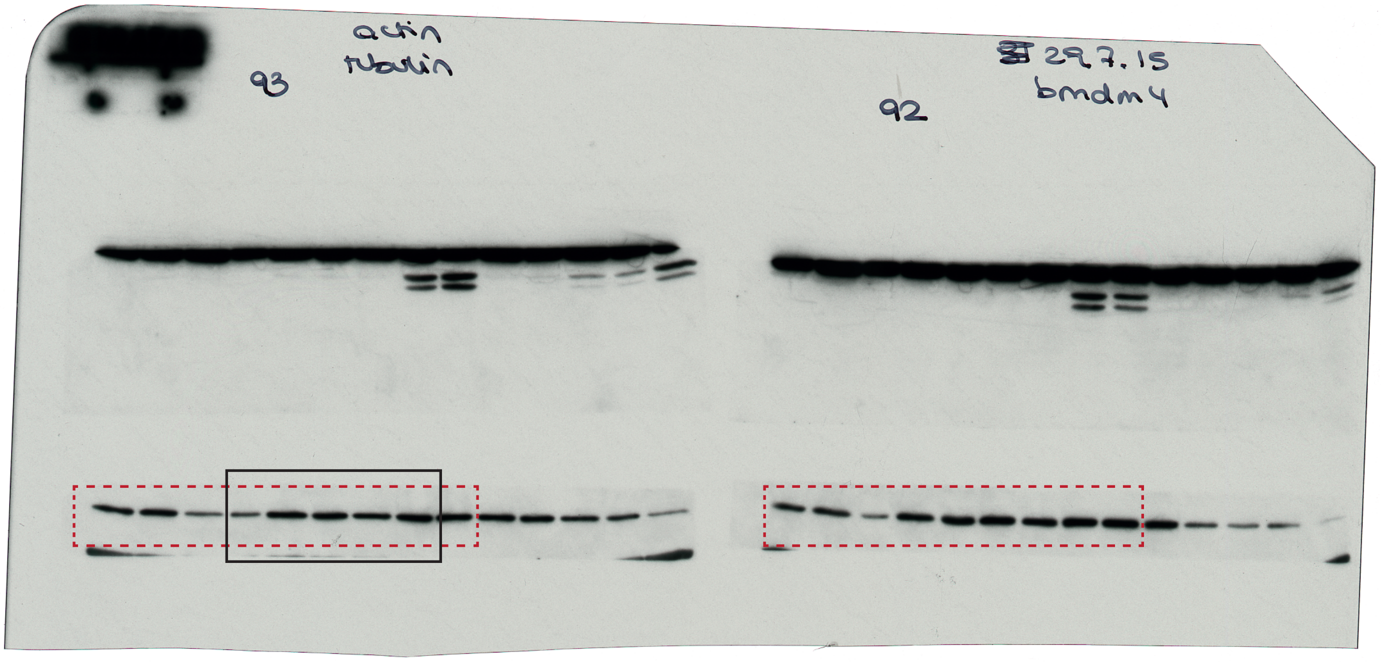

Figure 2 - figure supplement 1. Pannel A. CPEB4. Replicate 3

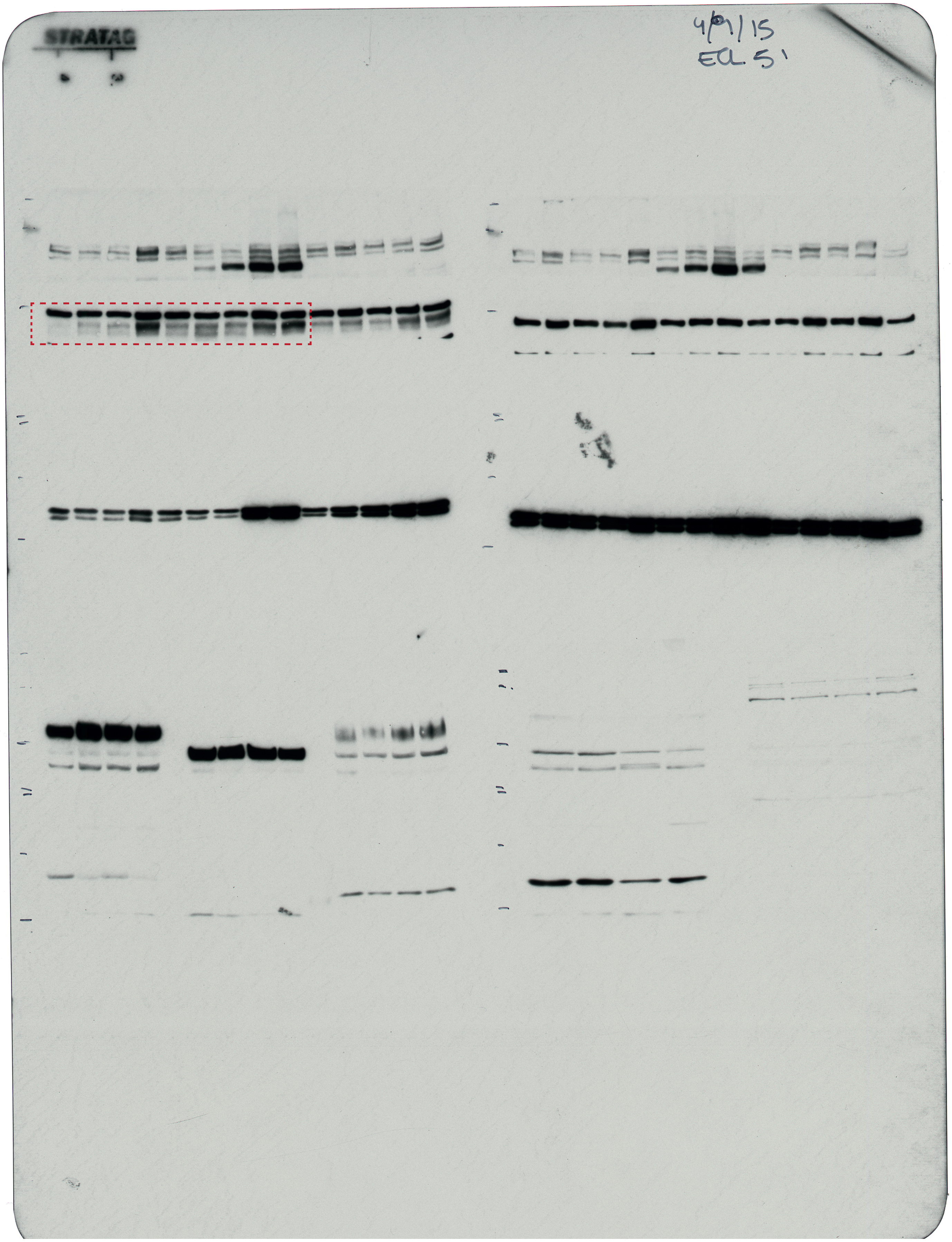

Figure 2 - figure supplement 1. Pannel A. Tubulin. Replicate 3

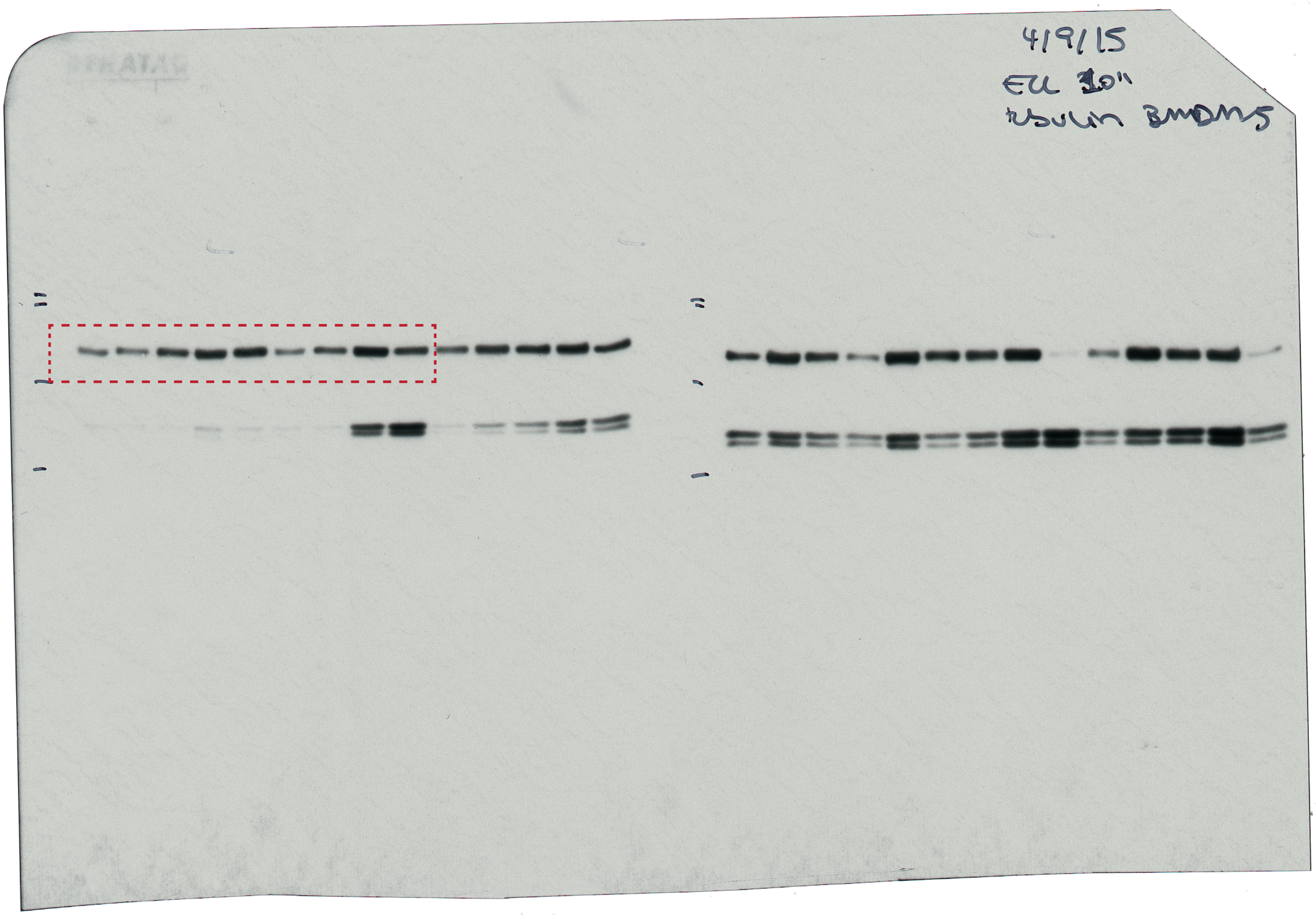

Supplement: Figure 2—source data 1. [file elife-75873-fig2-data1.pdf]
