# Supplementary material for: Macrophage inflammation resolution requires CPEB4-directed offsetting of mRNA degradation
Source: eLife. 2022 Apr 20;11:e75873. doi: 10.7554/eLife.75873 (PMC9094754; doi:10.7554/eLife.75873)

Figure 2C. CPEB4. Replicates 1 and 2.
Red boxes (Replicate 1): Not shown in figure, but used for quantification.
Black boxes (Replicate 2): Fig 2C.

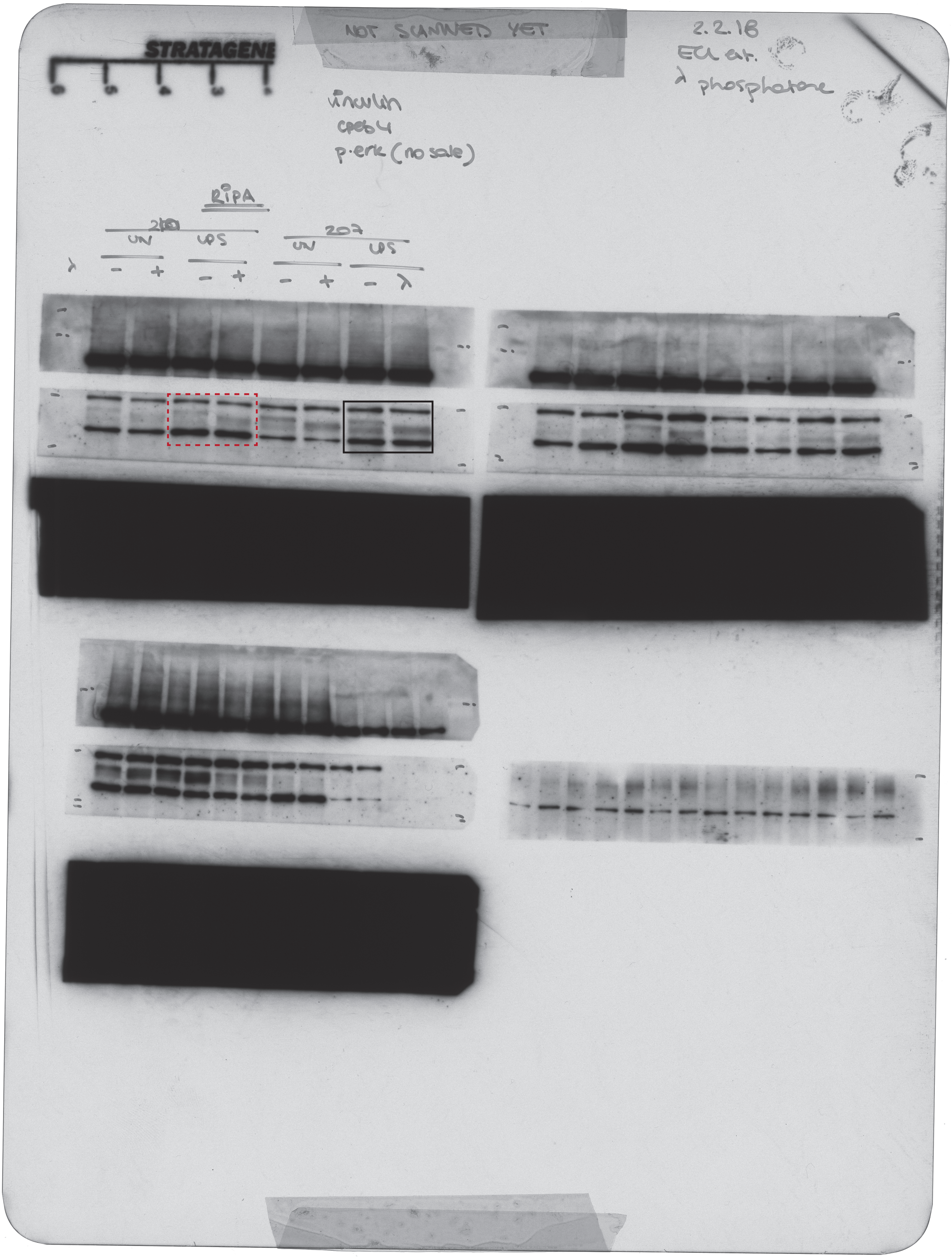

Figure 2C. Vinculin. Replicates 1 and 2.
Red boxes (Replicate 1): Not shown in figure, but used for quantification.
Black boxes Replicate 2): Fig 2C.

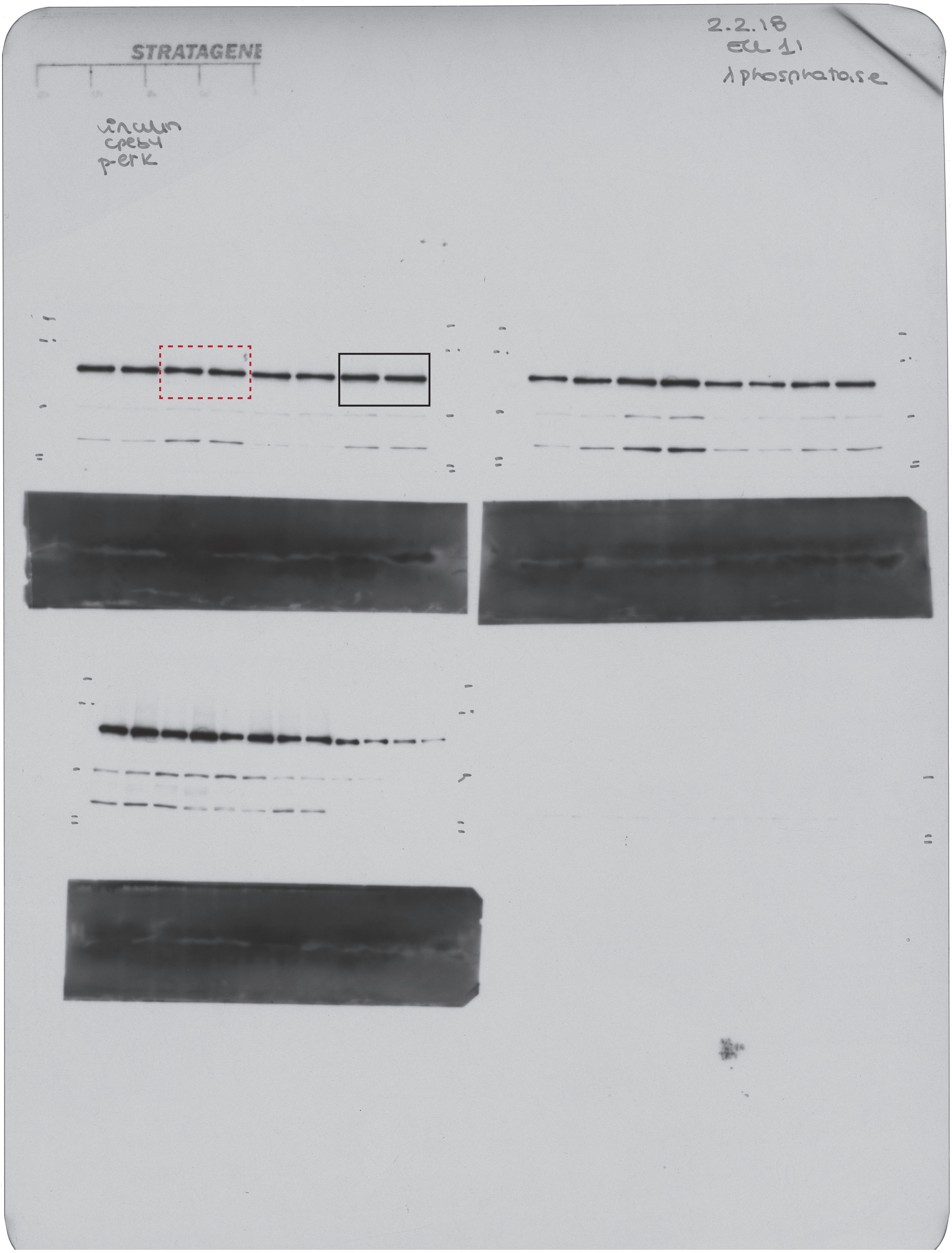

Figure 2C. Replicate 3 (not shown in figure, but used for quantification). CPEB4.

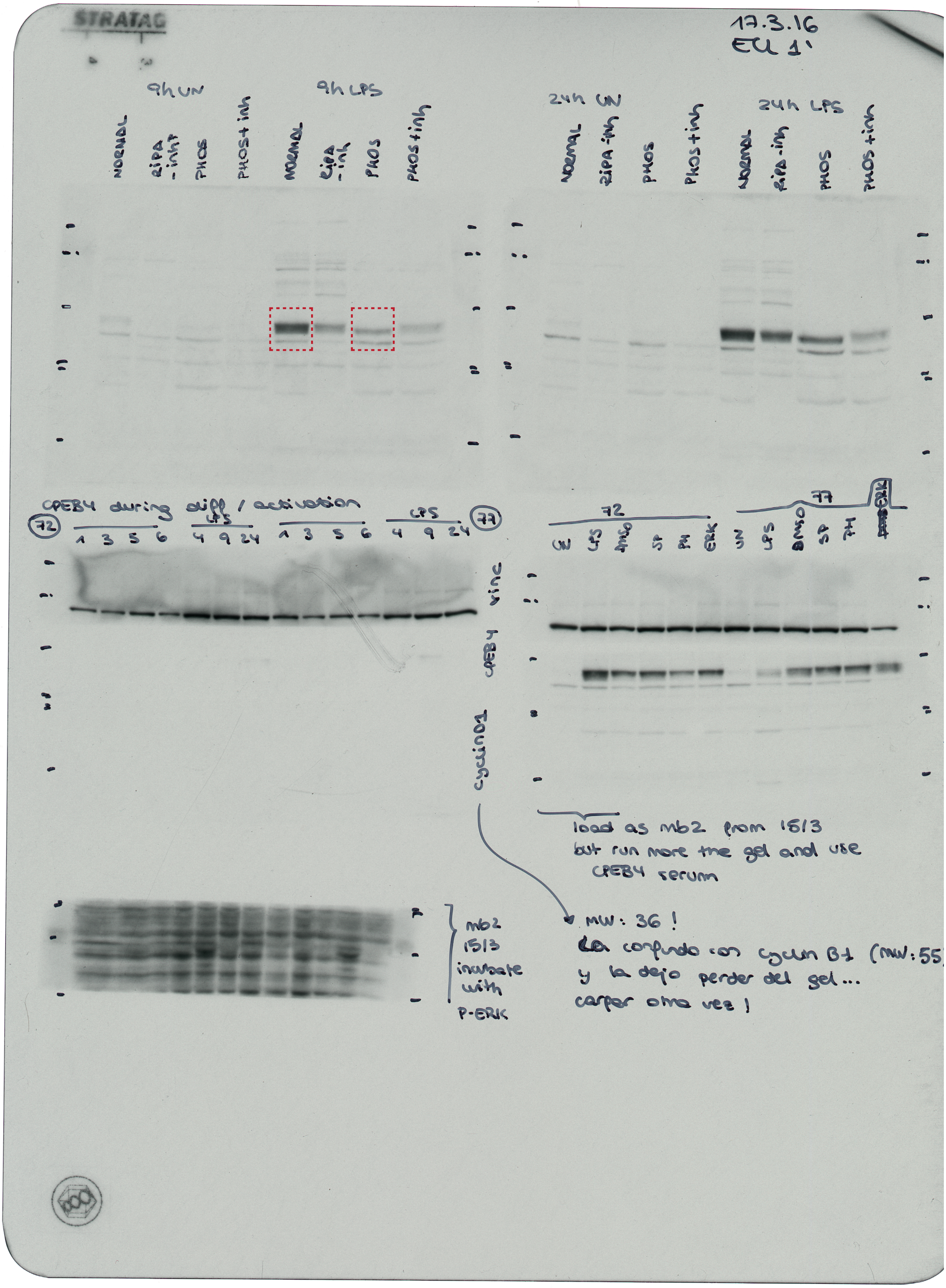

Figure 2C. Replicate 3 (not shown in figure, but used for quantification). Vinculin.

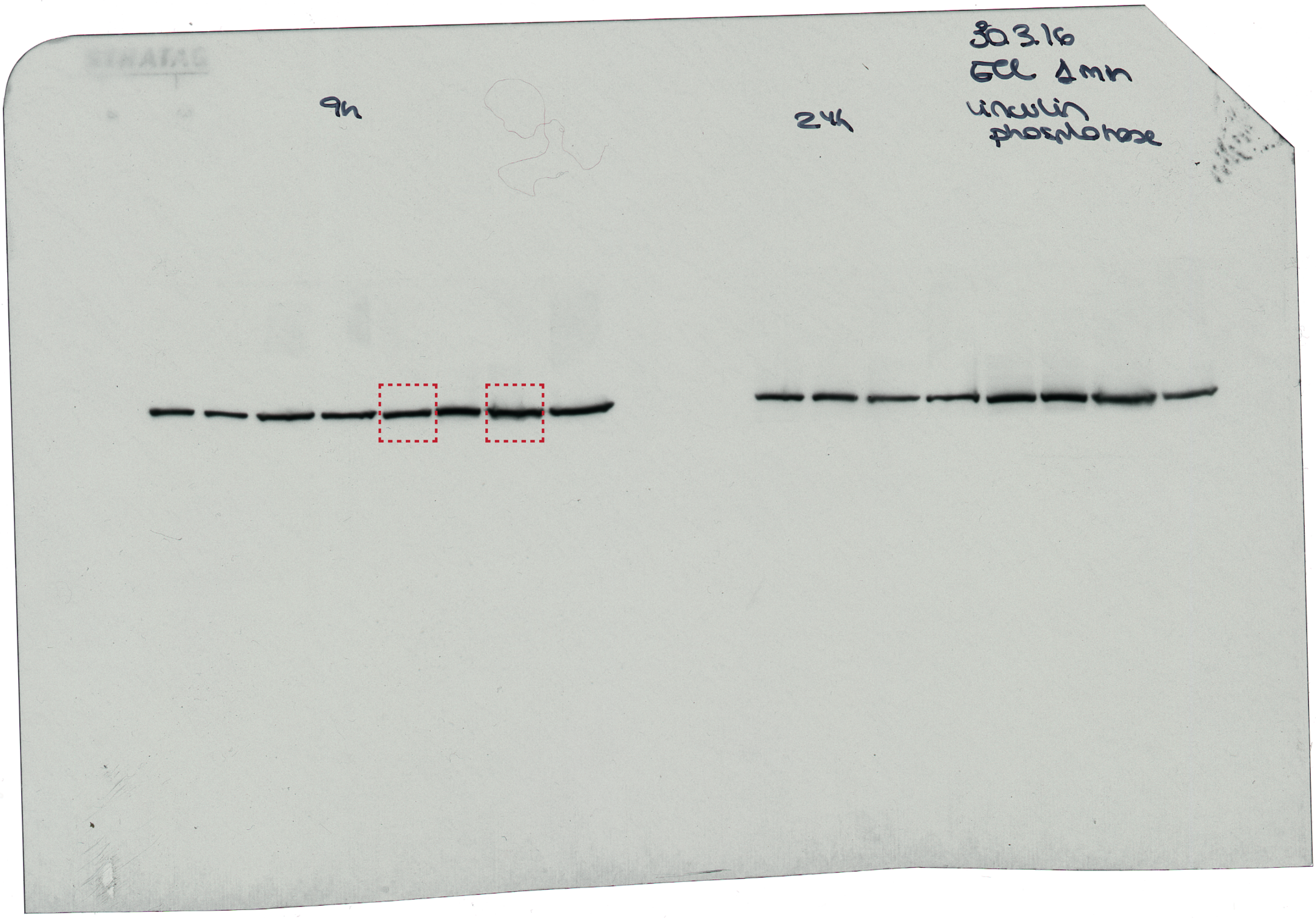

Supplement: Figure 2—source data 2. [file elife-75873-fig2-data2.pdf]
