# Supplementary material for: Macrophage inflammation resolution requires CPEB4-directed offsetting of mRNA degradation
Source: eLife. 2022 Apr 20;11:e75873. doi: 10.7554/eLife.75873 (PMC9094754; doi:10.7554/eLife.75873)

Black Box. Figure 2F. HIF1a Replicate 1 (also shown in Figure 2 - figure supplement 3).
Red Box. Figure 2 - figure supplement 3. HIF1a. Replicate 2.

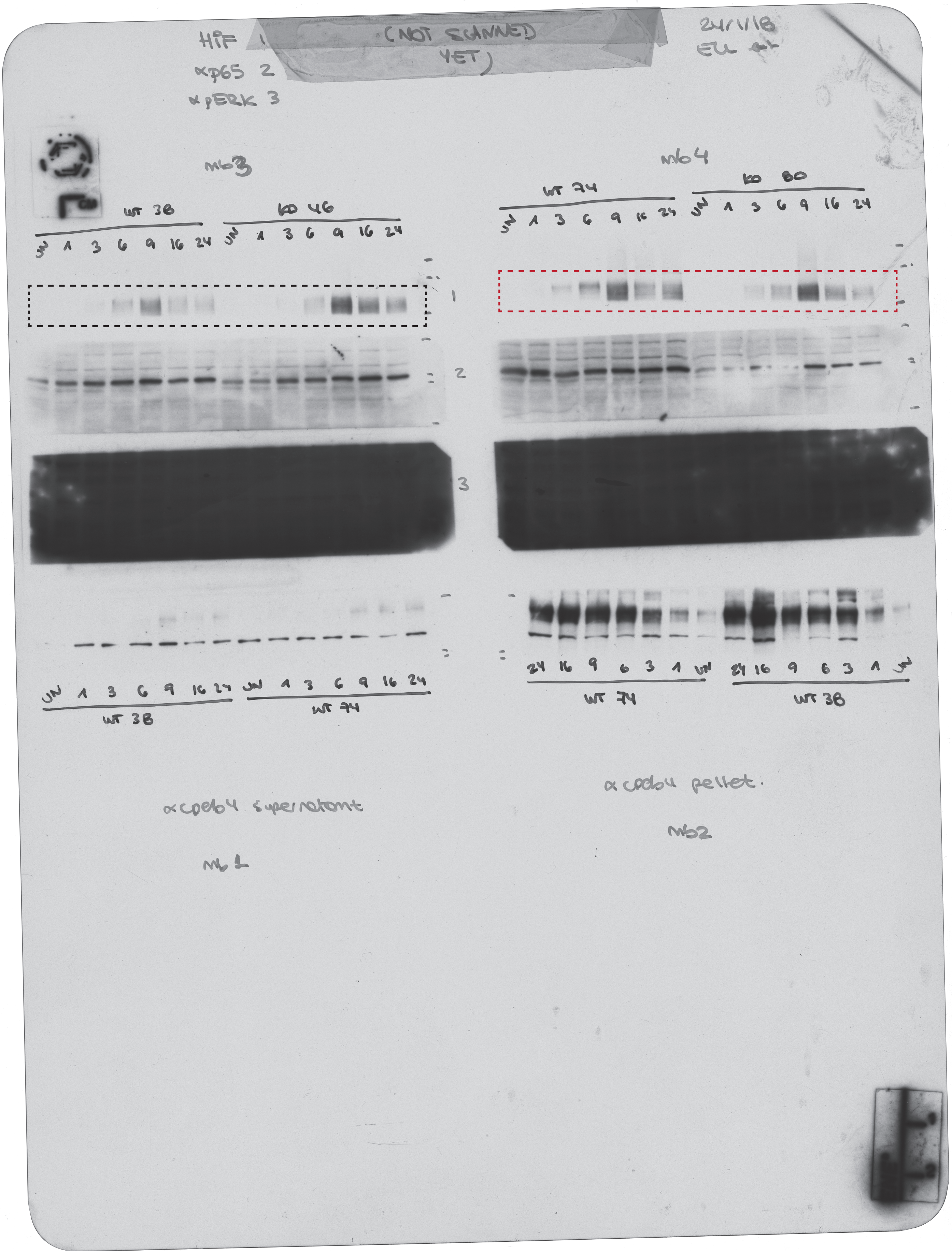

Black Box. Figure 2F. Vinculin Replicate 1 (also shown in Figure 2 - figure supplement 3).
Red Box. Figure 2 - figure supplement 3. Vinculin. Replicate 2.

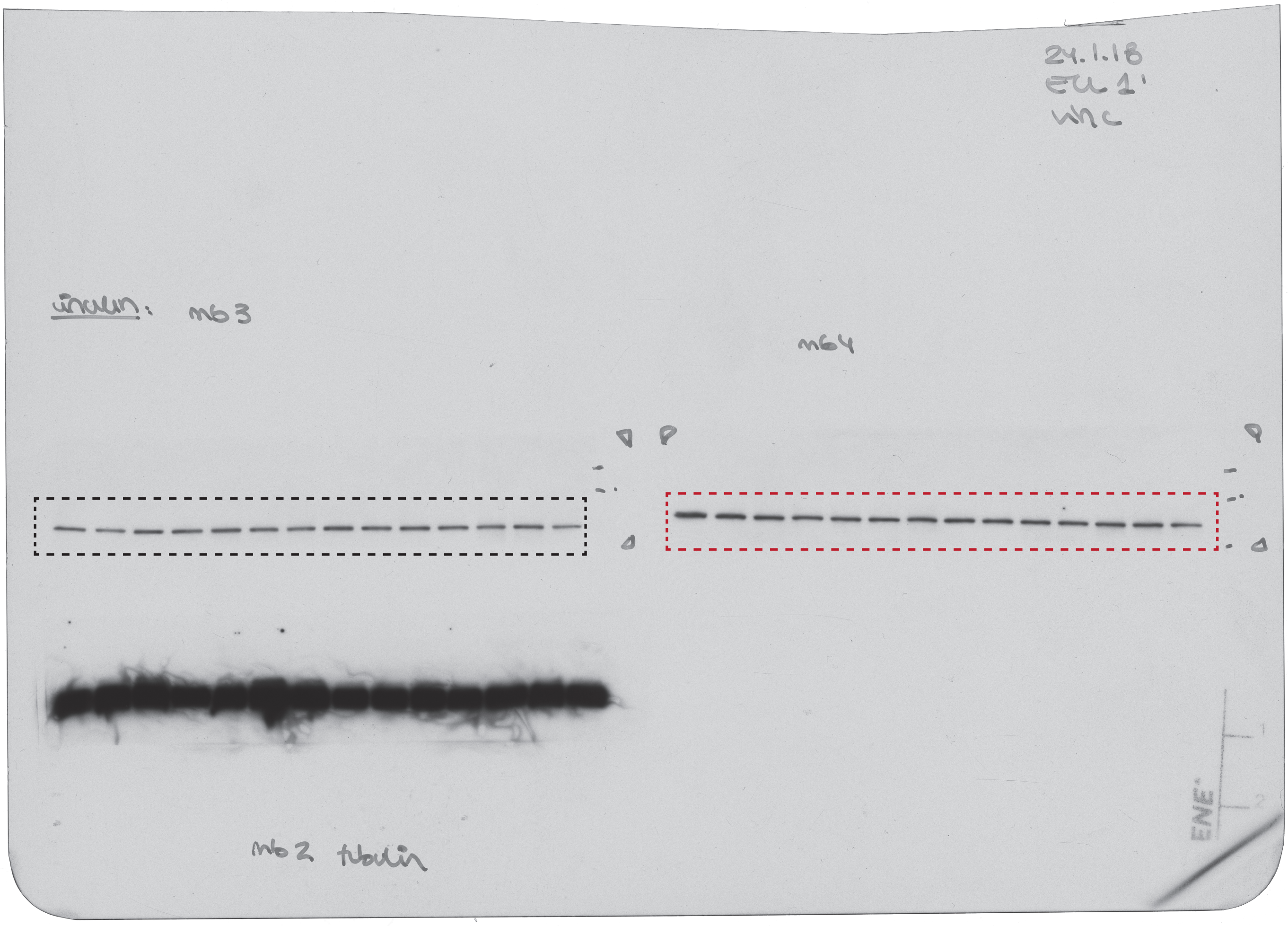

Black Box. Figure 2 - figure supplement 3. HIF1a. Replicate 3.

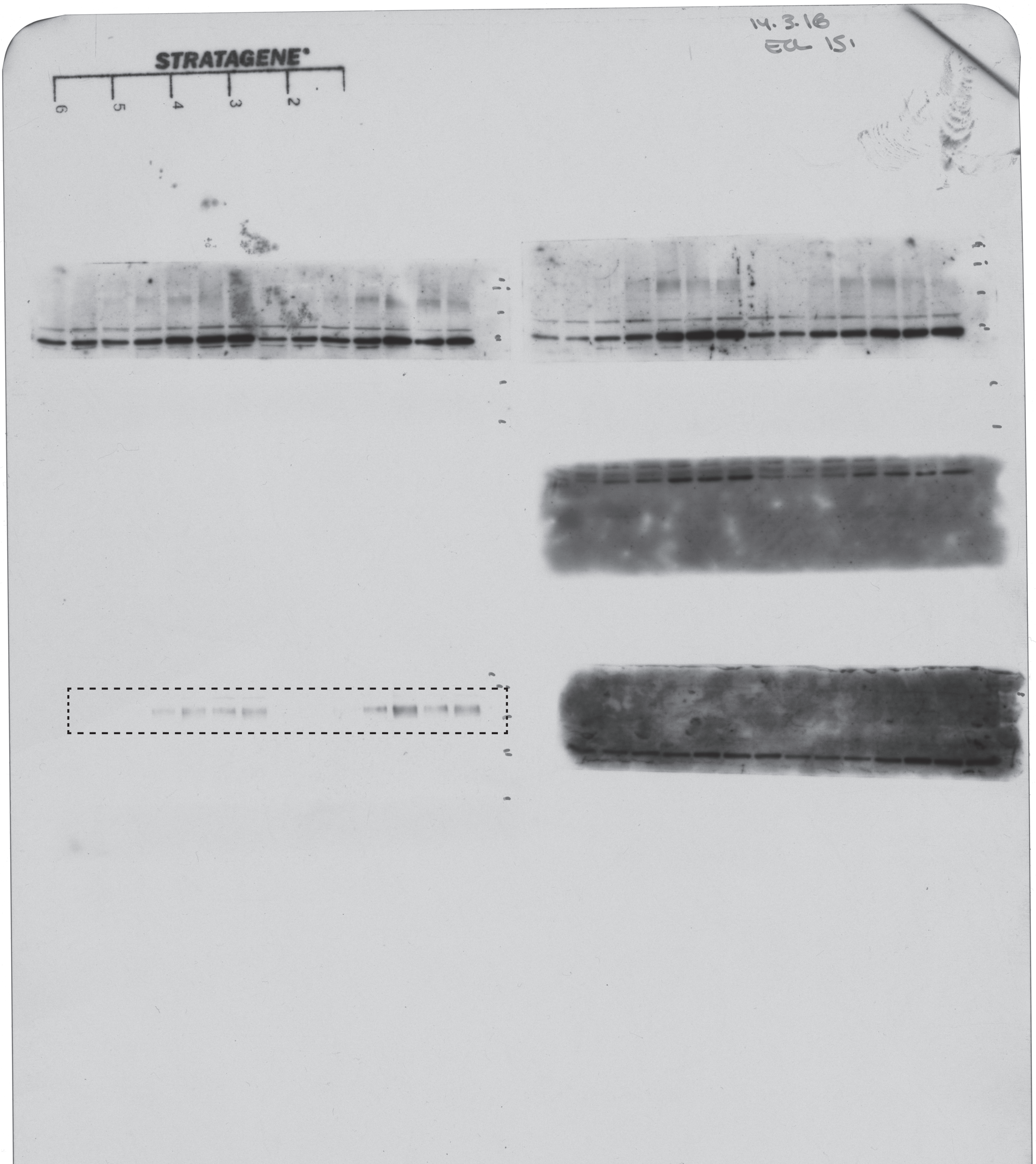

Black Box. Figure 2 - figure supplement 3. Vinculin. Replicate 3.

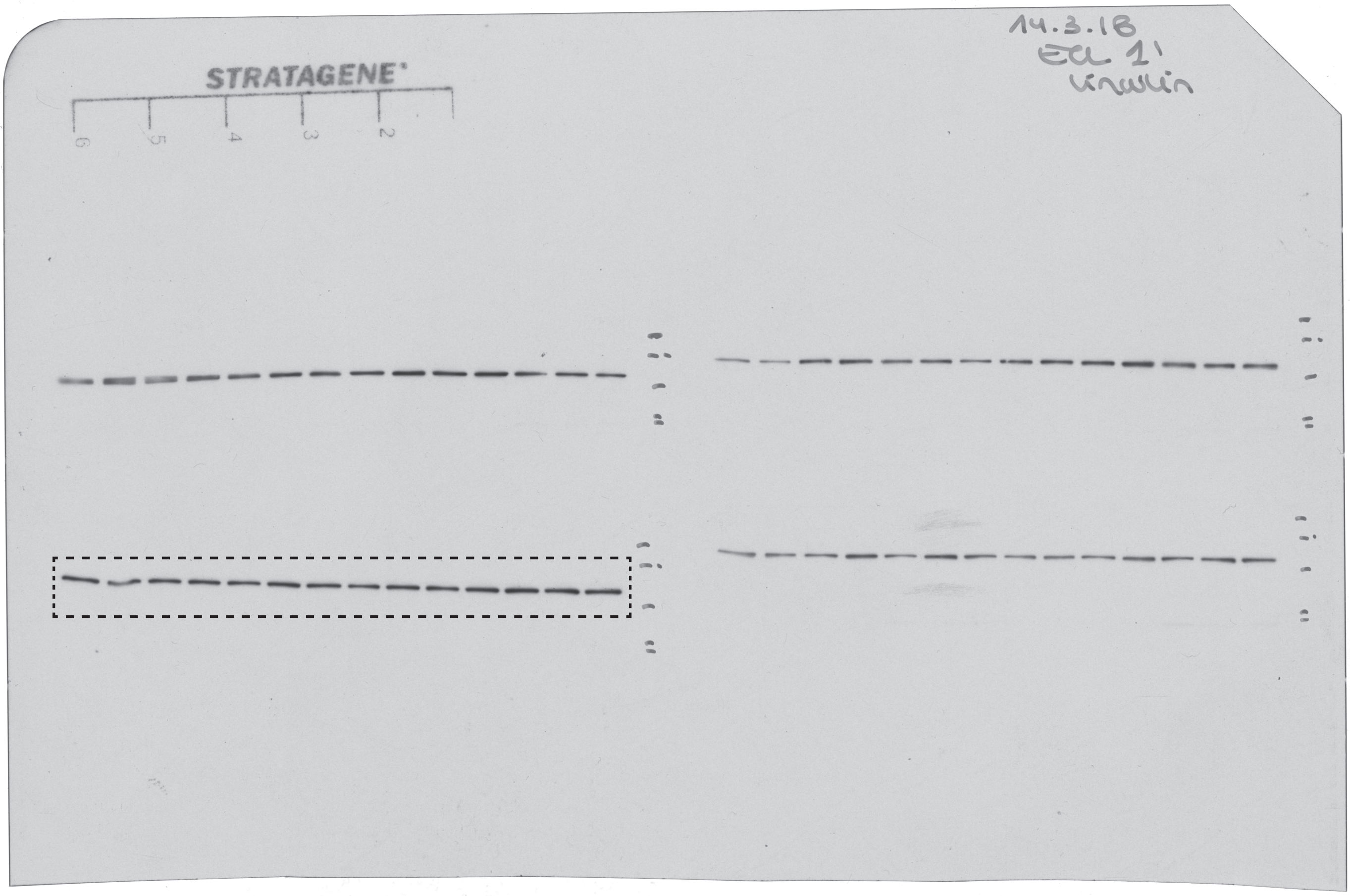

Supplement: Figure 2—source data 3. [file elife-75873-fig2-data3.pdf]
