# Supplementary figures and images for: Macrophage inflammation resolution requires CPEB4-directed offsetting of mRNA degradation
Source: eLife. 2022 Apr 20;11:e75873. doi: 10.7554/eLife.75873 (PMC9094754; doi:10.7554/eLife.75873)

Black Box. Figure 3E. TTP

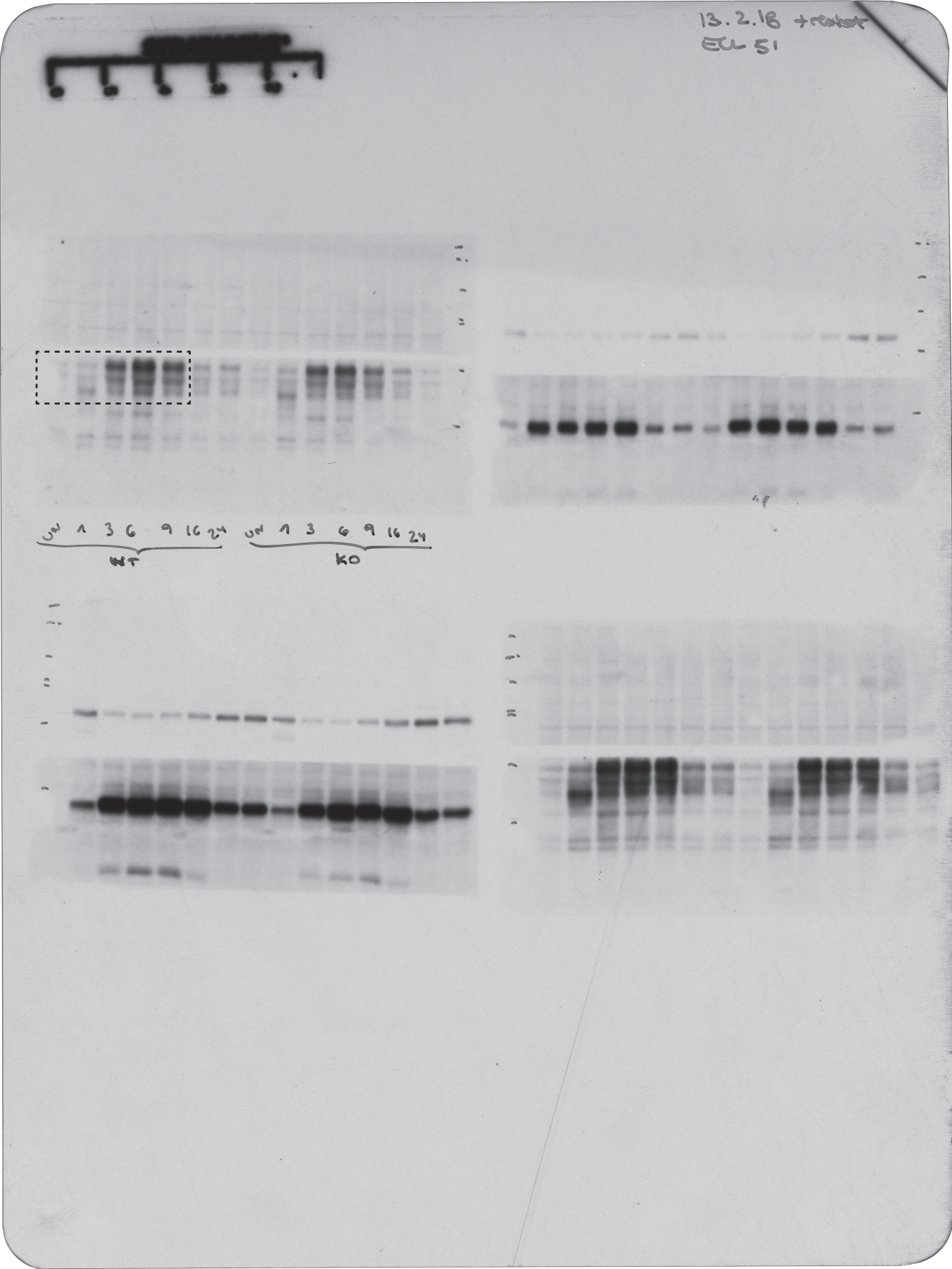

Black Box. Figure 3E. Vinculin

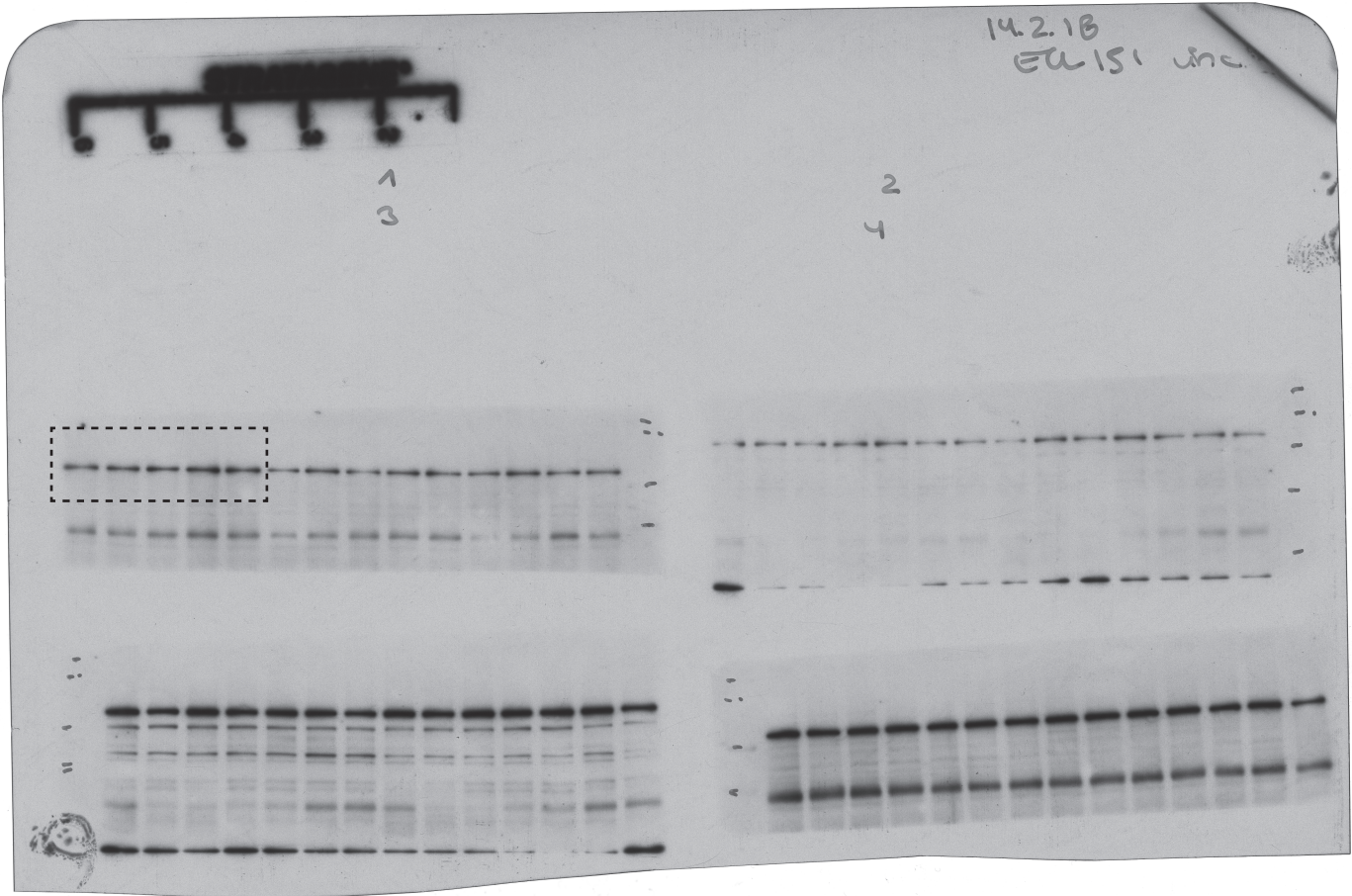

Supplement: Figure 3—source data 1. [file elife-75873-fig3-data1.pdf]

Black Box. Figure 4A. CPEB4

Red Box. Figure 4A. Vinculin

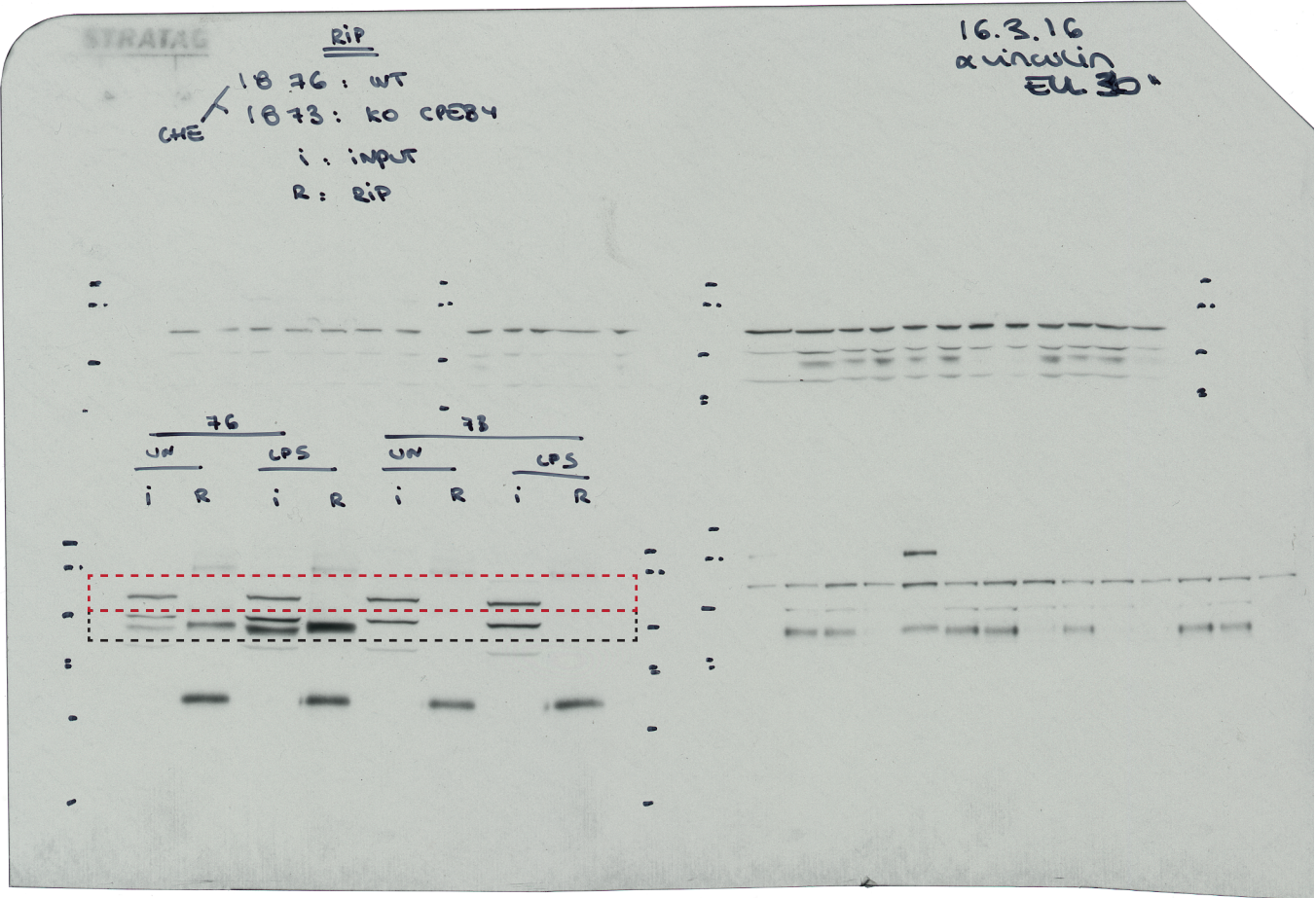

Supplement: Figure 4—source data 1. [file elife-75873-fig4-data1.pdf]
