# Supplementary material for: Macrophage inflammation resolution requires CPEB4-directed offsetting of mRNA degradation
Source: eLife. 2022 Apr 20;11:e75873. doi: 10.7554/eLife.75873 (PMC9094754; doi:10.7554/eLife.75873)

Black Box. Figure 4G. SOCS1 Replicate 1

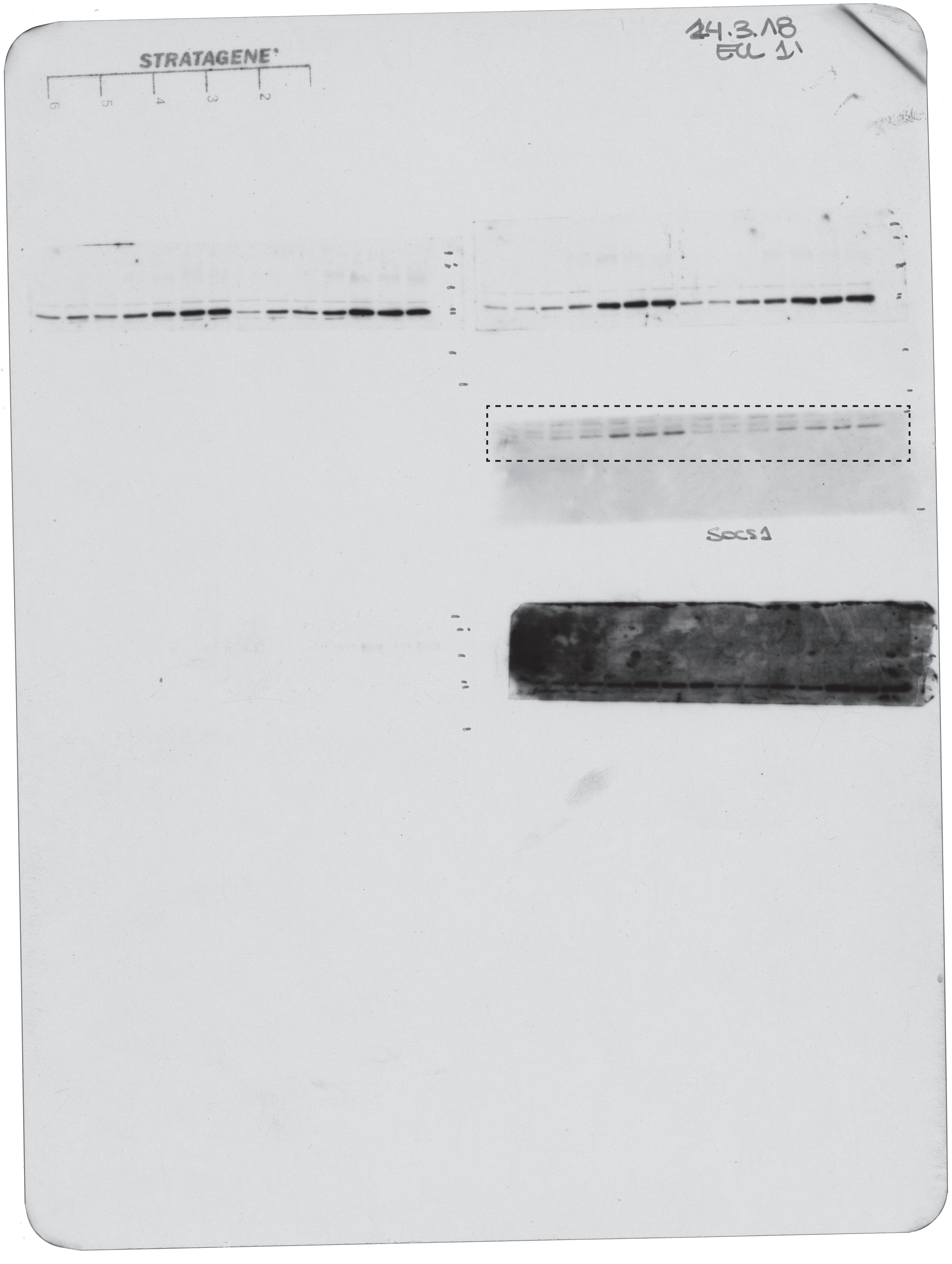

Black Box. Figure 4G. Vinculin. Replicate 1

Red and Blue Boxes. Figure 4G. Vinculin for SOCS1. Replicate 2 and 3.

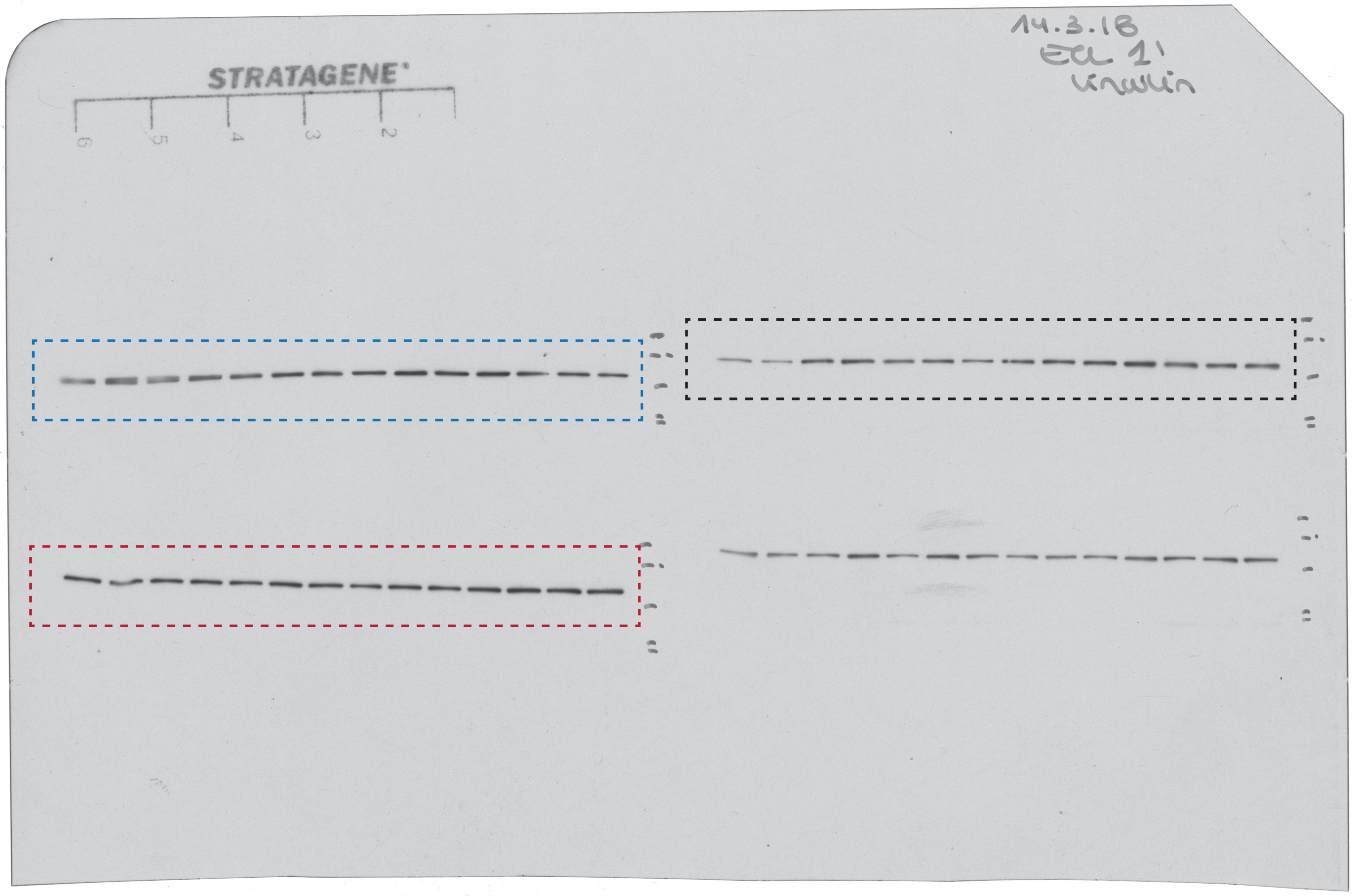

Red Box. Figure 4G. SOCS1 Replicate 2 (not shown in figure, but used for quantification)

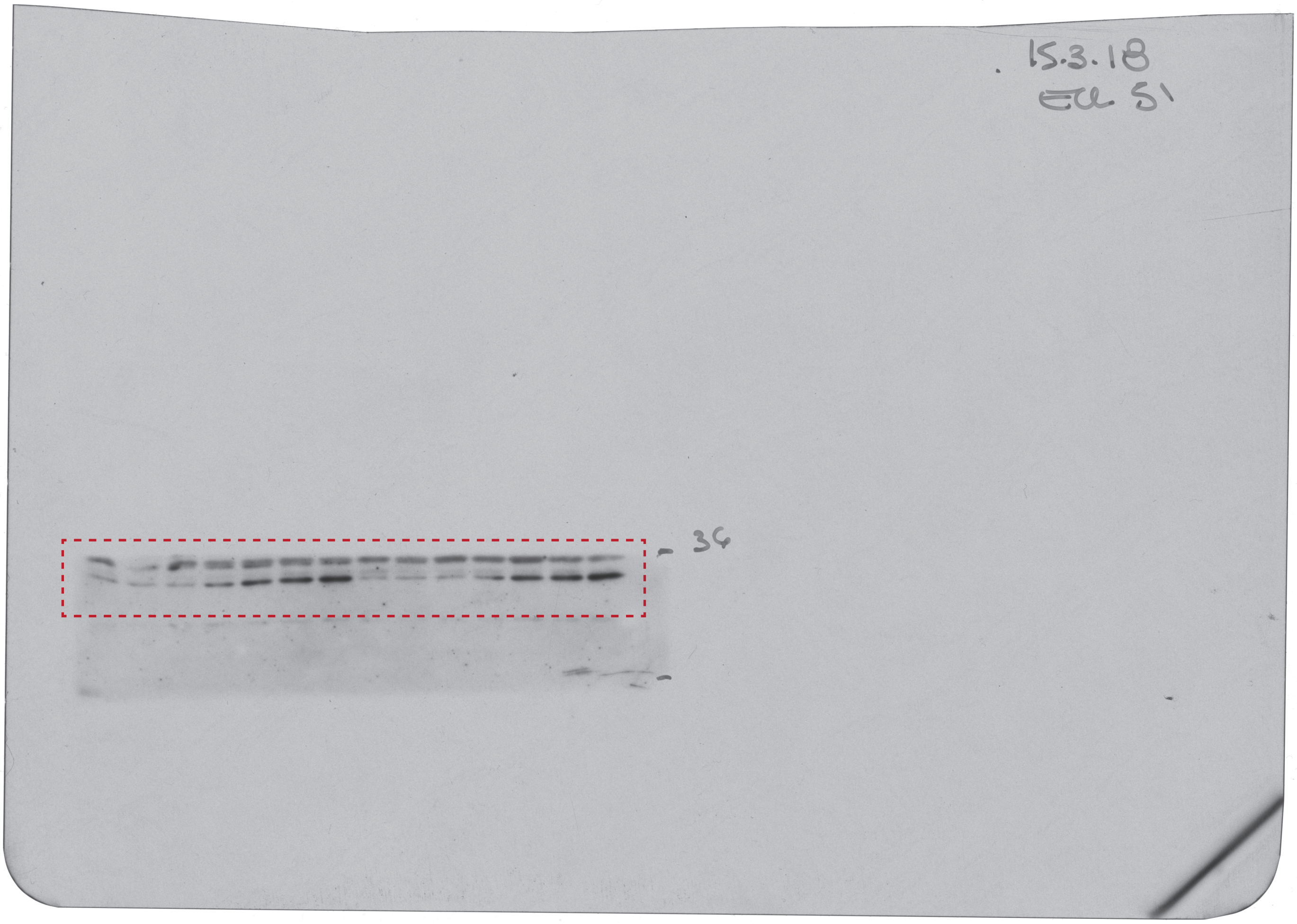

Blue Box. Figure 4G. SOCS1 Replicate 3 (not shown in figure, but used for quantification)

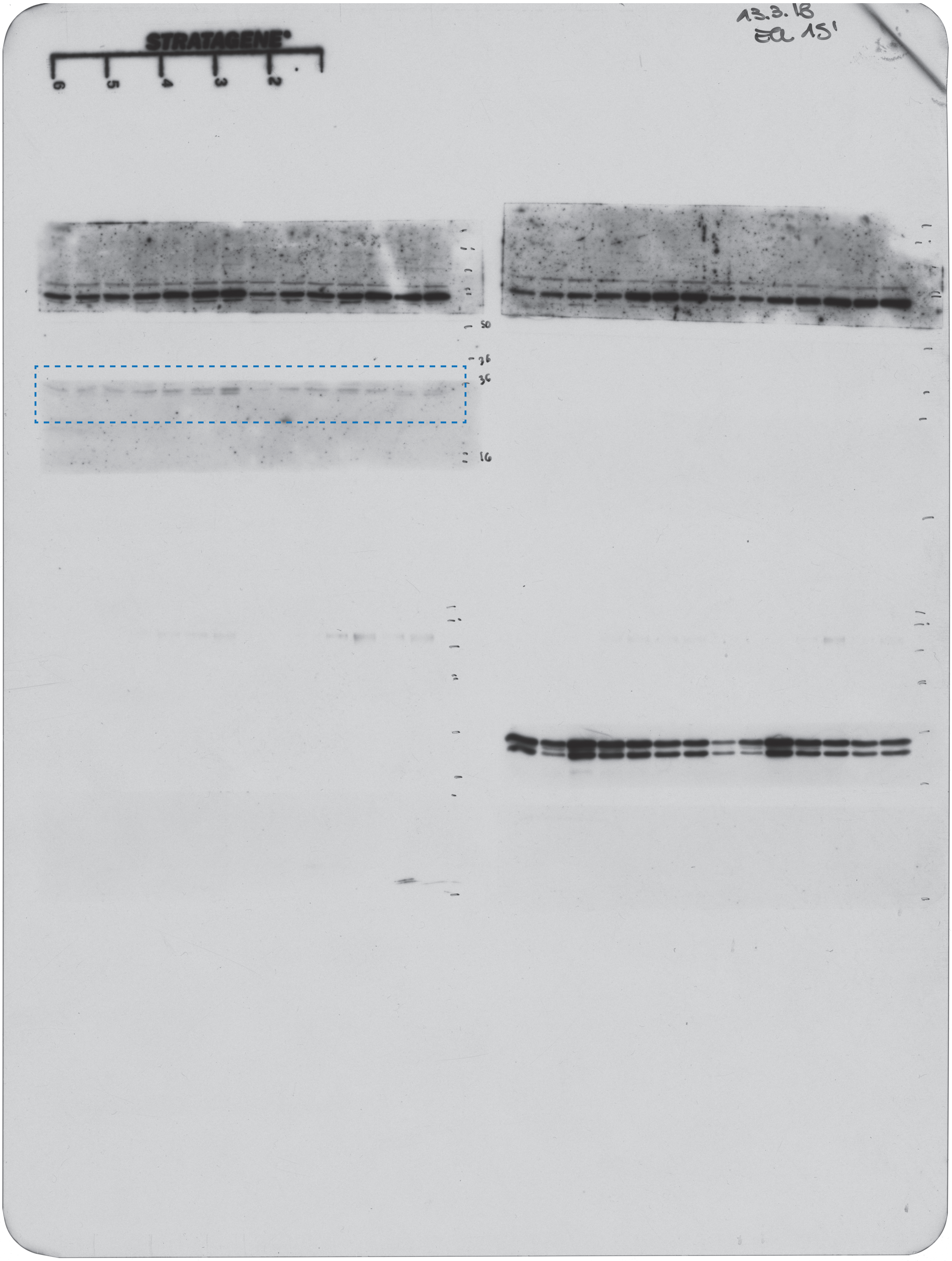

Supplement: Figure 4—source data 2. [file elife-75873-fig4-data2.pdf]
