# Supplementary material for: Macrophage inflammation resolution requires CPEB4-directed offsetting of mRNA degradation
Source: eLife. 2022 Apr 20;11:e75873. doi: 10.7554/eLife.75873 (PMC9094754; doi:10.7554/eLife.75873)

Black Box. Figure 6B. P-ERK

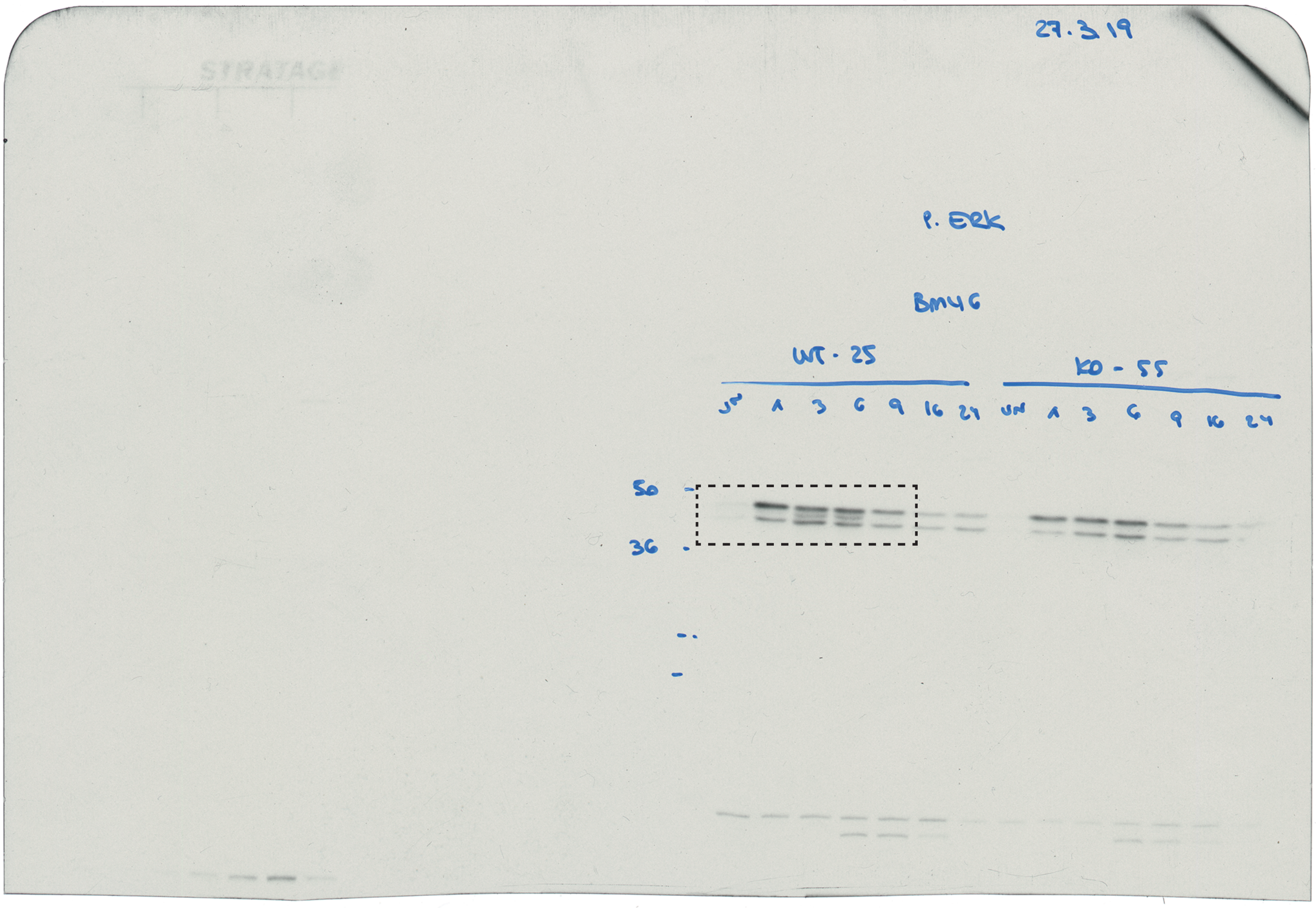

Black Box. Figure 6B. Vinculin for P-ERK

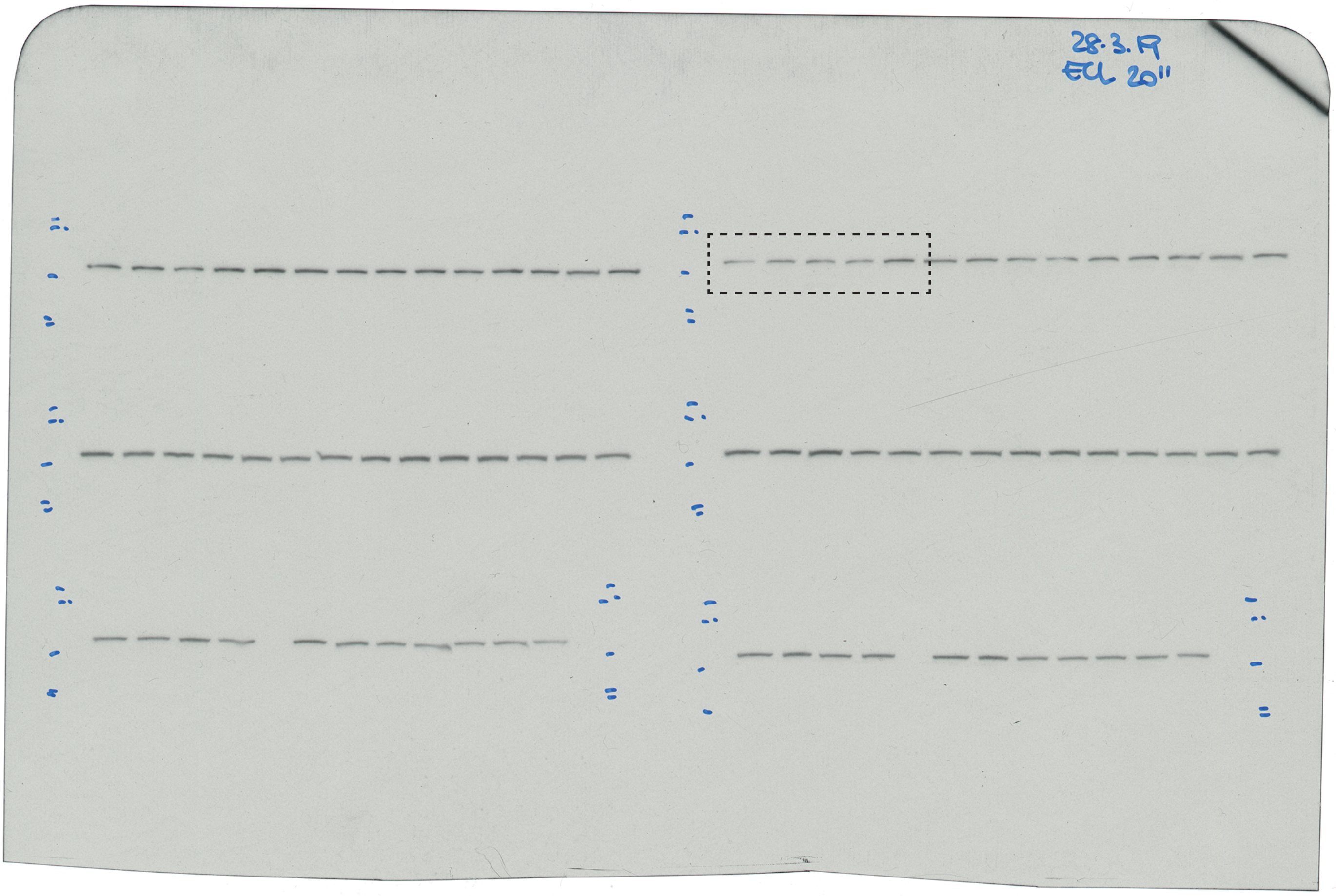

Black Box. Figure 6B. CPEB4

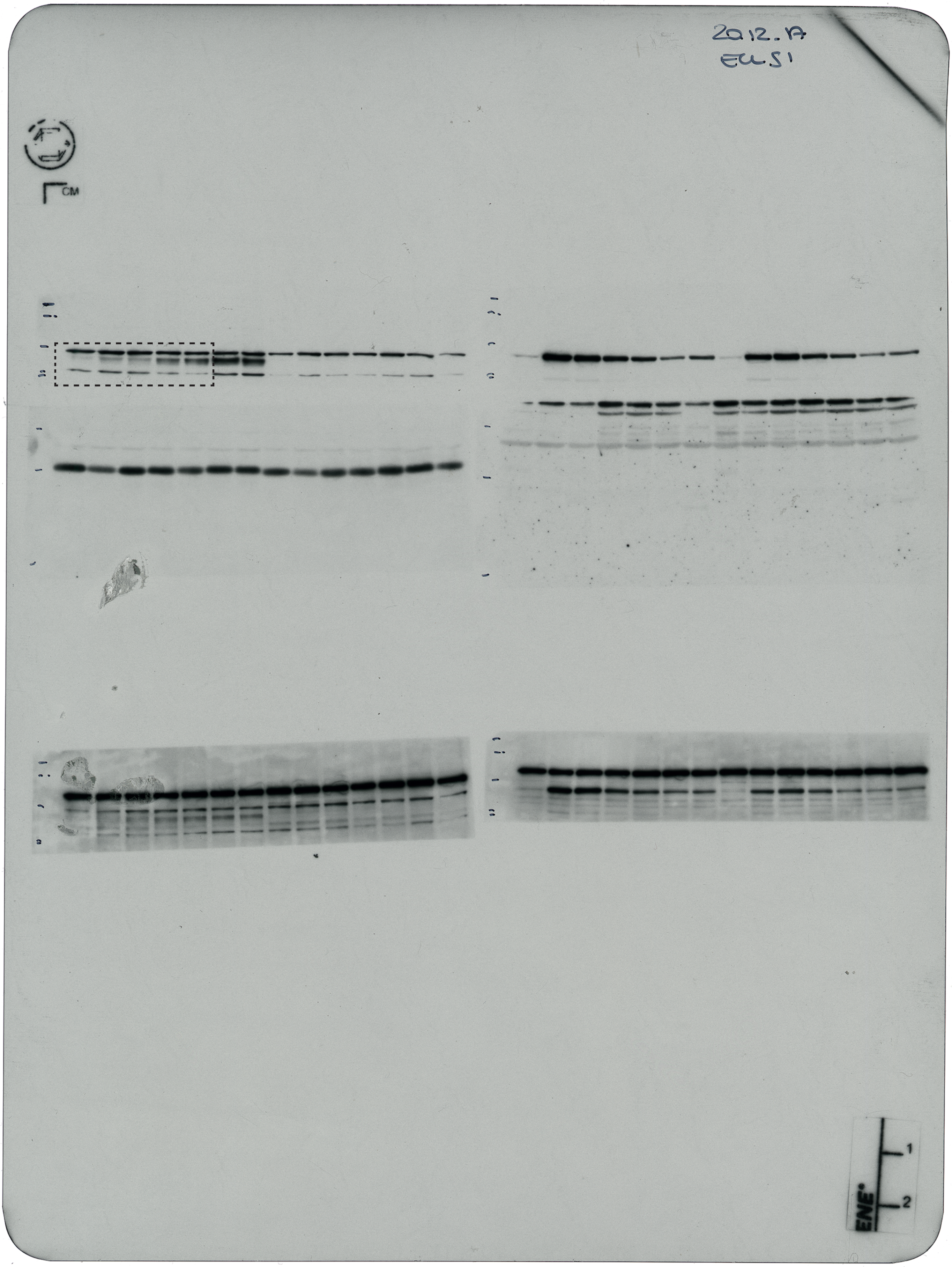

Black Box. Figure 6B. Vinculin for CPEB4

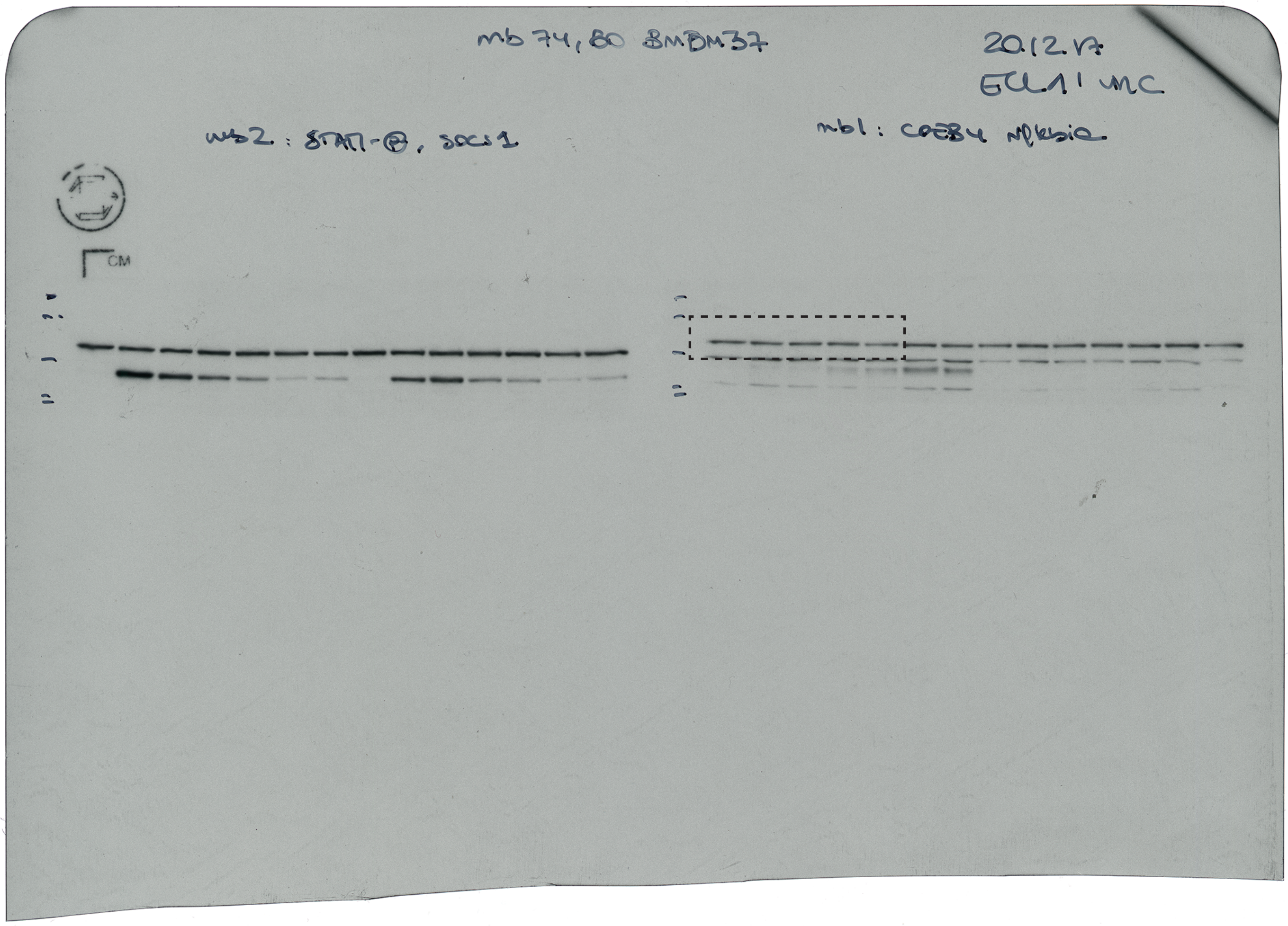

Black Box. Figure 6B. TTP

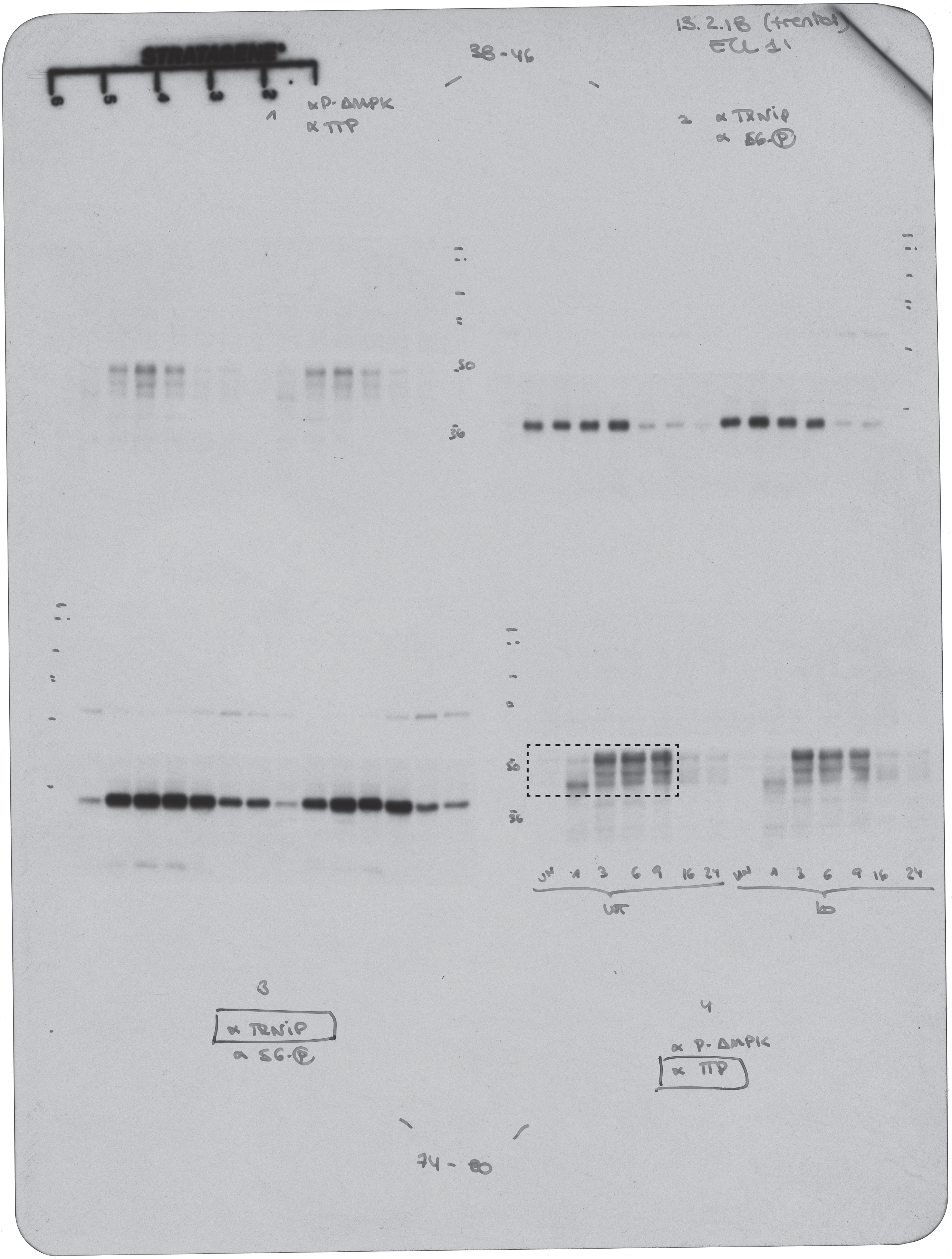

Black Box. Figure 6B. Vinculin for TTP

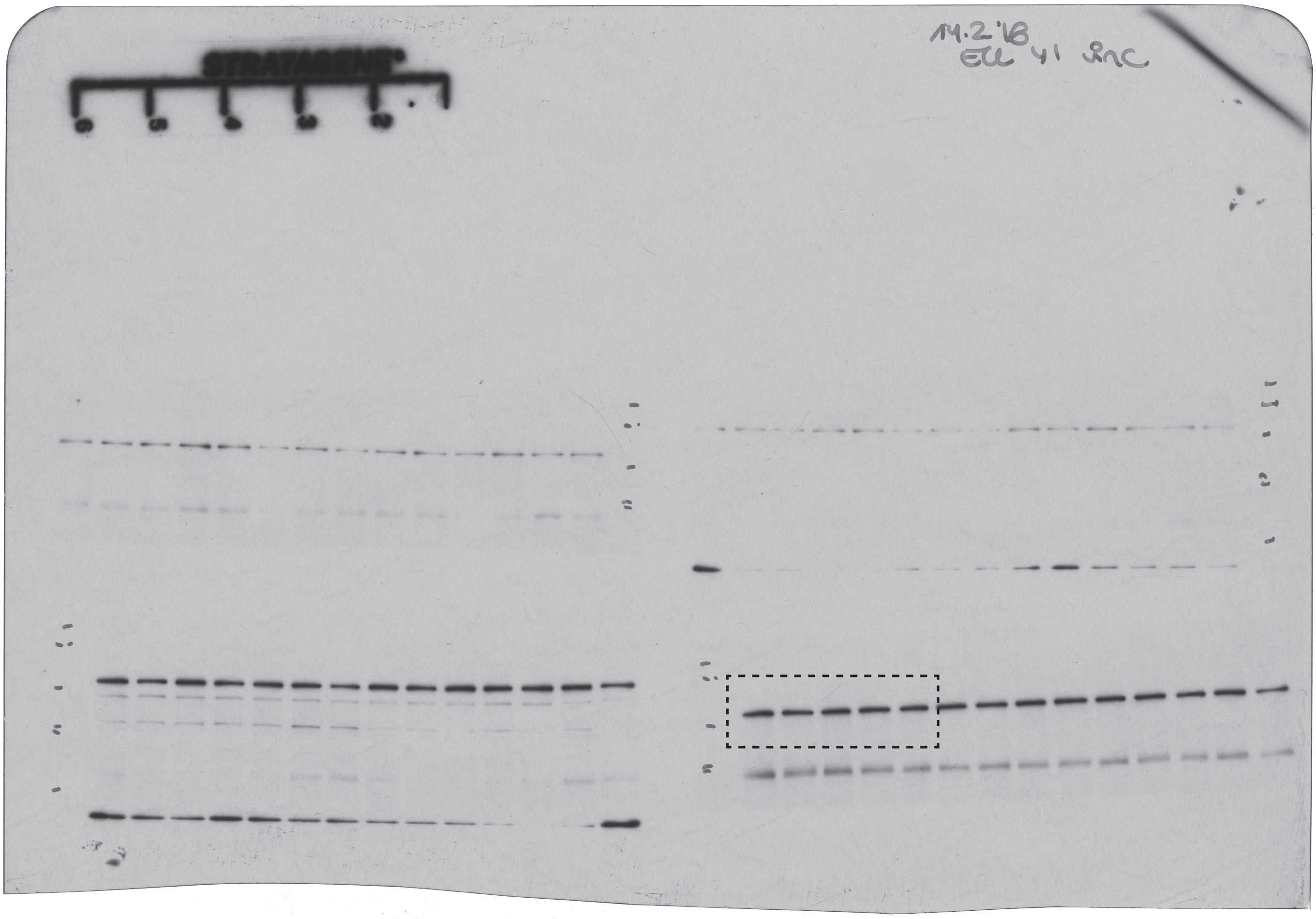

Black Box. Figure 6B. P-MK2 and Ponceau Staining

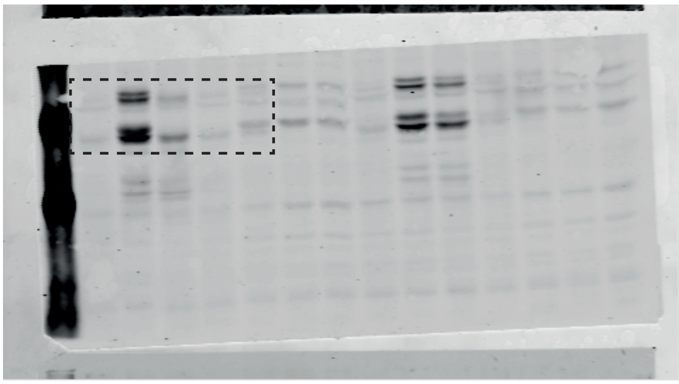

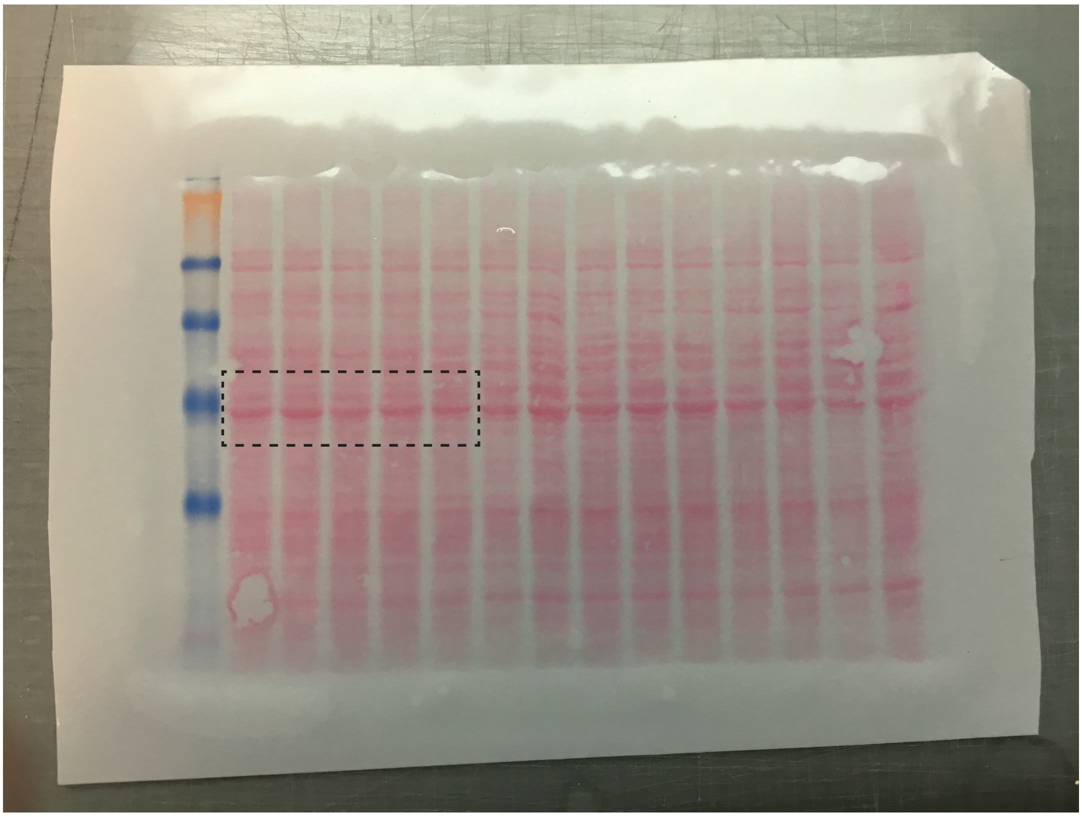

Black Box. Figure 6B. P-p38 and Ponceau Staining

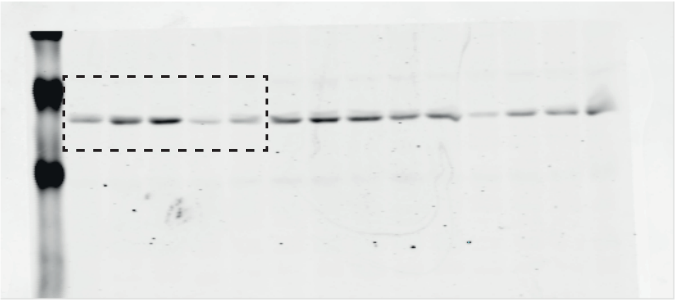

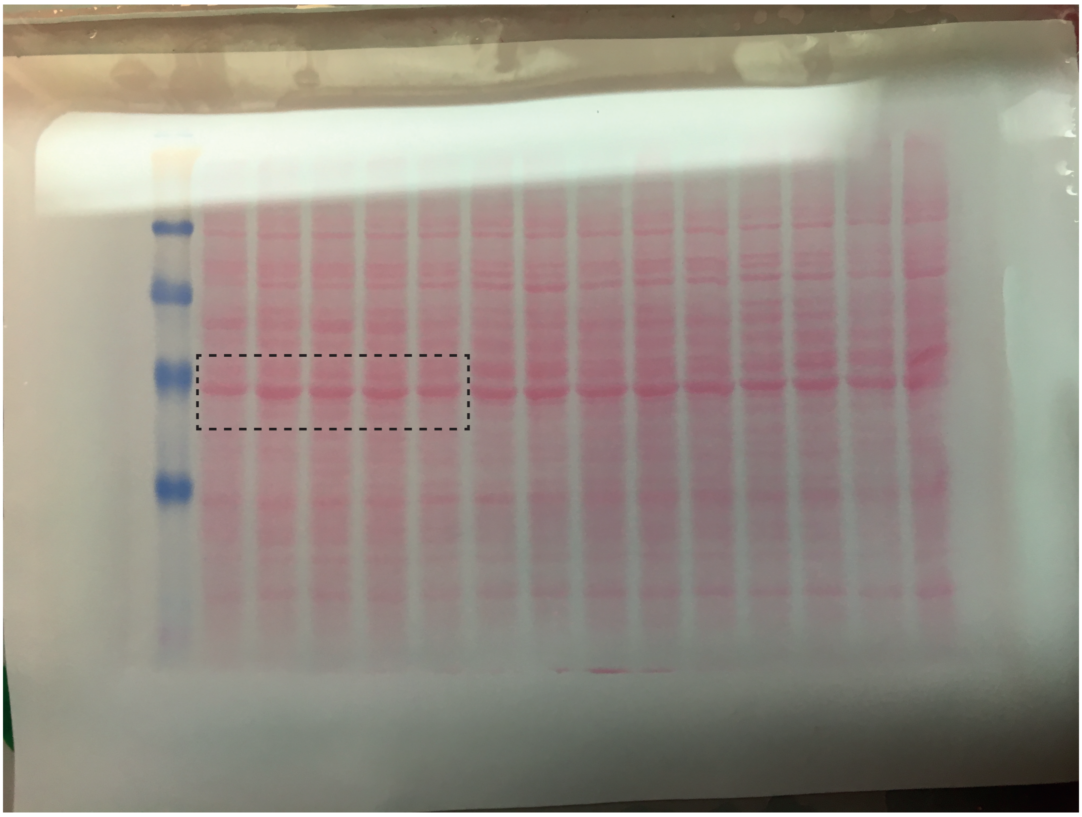

Supplement: Figure 6—source data 1. [file elife-75873-fig6-data1.pdf]
